# Analysis of the mediating role of BMI in associations of different folate forms with hepatic steatosis and liver fibrosis in adolescents in the USA: results from the NHANES 2017-2018

**DOI:** 10.3389/fendo.2023.1273580

**Published:** 2023-12-05

**Authors:** Jingli Wen, Yuanyuan Fei, Ling Yuan, Kai Li, Qian Xu, Xueyan Cao, Jing Su, Yujing Zhu, Zhenjiang Zhang

**Affiliations:** ^1^ Department of Infection, The Affiliated Suqian first people's Hospital of Nanjing Medical University, Suqian, JiangSu, China; ^2^ Department of Infection, The Affiliated Zhangjiagang Hospital of Soochow University, Suqian, JiangSu, China; ^3^ Department of Infection, The Affiliated Lianyungang Hospital of Xuzhou Medical University, Suqian, JiangSu, China; ^4^ Laboratory of Department of hematology, The Affiliated Suqian first people's Hospital of Nanjing Medical University, Suqian, JiangSu, China; ^5^ Clinical Research Center, The Affiliated Suqian first people's Hospital of Nanjing Medical University, Suqian, JiangSu, China

**Keywords:** folate, hepatic steatosis, liver fibrosis, BMI, NAFLD, significant fibrosis

## Abstract

**Background:**

Most studies have explored the relationship between serum total folate and nonalcoholic fatty liver disease (NAFLD) in adults, but there has been no study on the relationship between different folate forms and hepatic steatosis or liver stiffness in adolescents.

**Objective:**

To investigate the association of different folate forms with hepatic steatosis or liver stiffness in adolescents, and further explore the intermediary role of BMI in this relationship.

**Methods:**

The cross-sectional study included 549 participants from the 2017-2018 National Health and Nutrition Inspection Survey (NHANES) survey cycle who had complete data. Four folate data (red blood cell folate, serum total folate, 5-methyl-tetrahydrofolate and folic acid) were included in our study. Controlled attenuation parameters (CAP) and liver stiffness came from the results of liver ultrasound transient elastography. We used linear regression to analyze the relationship between different forms of folate and CAP or liver stiffness, and logistic regression to analyze the relationship between different forms of folate and NAFLD or significant fibrosis. We also used restricted cubic splines to analyze the nonlinear relationship between different forms of folate and NAFLD or significant fibrosis. Finally, we used regression-based intermediary analysis to distinguish the direct and BMI-mediated effects of folate on CAP or liver stiffness. All the analyses adjusted the relevant covariates.

**Results:**

The means of CAP and liver hardness in this study were 223.02dB/m and 5.03kPa, respectively. We found that in model 2, there was a negative correlation between serum total folate (β: -18.53; 95%CI: -29.32 to -7.73) or 5-methyltetrahydrofolate (β: -14.13; 95%CI: -28.98 to -7.86) and CAP. However, when the BMI was further adjusted in model 3, this negative correlation no longer existed (serum total folate: β: -8.36; 95%CI: -17.69 to 0.97; 5-methyltetrahydrofolate: β: -8.05; 95%CI: -17.19 to 1.09). Similarly, we found a negative correlation between serum total folate or 5-Methyl-tetrahydrofolate and liver stiffness in model 2. There was no significant correlation between red blood cell folate or folic acid and CAP or liver stiffness in either model 2 or model 3. The nonlinear relationship between different folate forms and NAFLD or significant fibrosis was not significant. It is estimated that 76% of the total association between serum total folate and CAP is mediated by BMI. The mediating proportion of BMI in the total correlation between serum total folate and liver stiffness was 50%. Similarly, we found that BMI significantly mediated the relationship between 5-Methyl-tetrahydrofolate and CAP or liver stiffness, with a mediating ratio of 77% and 49%, respectively.

**Conclusion:**

Our results show that serum total folate or 5-Methyl-tetrahydrofolate are negatively correlated with hepatic steatosis or liver stiffness in adolescents, and BMI plays major mediating role in this relationship. Our findings emphasize the importance of monitoring the concentration of serum folate, not just the serum total folate concentration.

## Introduction

Obesity is a major public health problem, with an obesity rate of 20.9% among adolescents aged 12-19 years in the United States (1). The prevalence of obesity has increased among all young people over the past few decades, leading to fatty liver disease becoming the most common liver disease in the U.S. adolescent population (2). Fatty liver disease is known to affect the long-term health of the liver and can lead to liver fibrosis and cirrhosis in adolescents (3). Despite the emergence of fatty liver as a public health problem, adolescent fatty liver has not been fully diagnosed due to lack of screening and awareness of potential long-term complications among health care providers (4, 5). Instantaneous elastography may be a useful tool for assessing adolescents at high risk for fatty liver disease due to obesity or other components of metabolic syndrome. Instantaneous elastography can simultaneously measure liver stiffness and control attenuation parameters (CAP), which has a good diagnostic accuracy for early detection of liver diseases in high-risk adolescents (6, 7).

At present, weight loss is still an important way to prevent nonalcoholic fatty liver disease (NAFLD). But losing weight is challenging to achieve and even more challenging to maintain, with only 20% of obese people able to do so (8). Therefore, drug approaches are being actively sought to reverse liver steatosis. Folate is a water-soluble vitamin B9 that plays an important role in single-carbon metabolism and methylation reactions (9). Some animal studies have shown that folate supplementation can reduce liver steatosis (10, 11). The blood contains various forms of folate, including 5-methyl-tetrahydrofolate, folic acid, 5-formyl-tetrahydrofolate, tetrahydrofolate and 5,10-methenyl-tetrahydrofolate. A case-control study showed that severe NAFLD in obese subjects was associated with lower serum folate concentrations (12). Two cross-sectional studies have shown that elevated serum folate may be negatively correlated with NAFLD in adults (13, 14). Only one cross-sectional study explored the relationship between different forms of folate and NAFLD in adults (15). To our knowledge, no current studies have investigated the relationship between different folate forms and CAP or liver stiffness in the adolescent population. Furthermore, several studies have shown that lower serum folate concentrations are associated with higher body mass index (BMI) (16–18).

Therefore, the aim of this study was to explore the relationship between the concentration of different forms of folate and CAP or liver stiffness in adolescents and the mediating role of BMI in this relationship.

## Methods

### Study population

**Figure 1:**
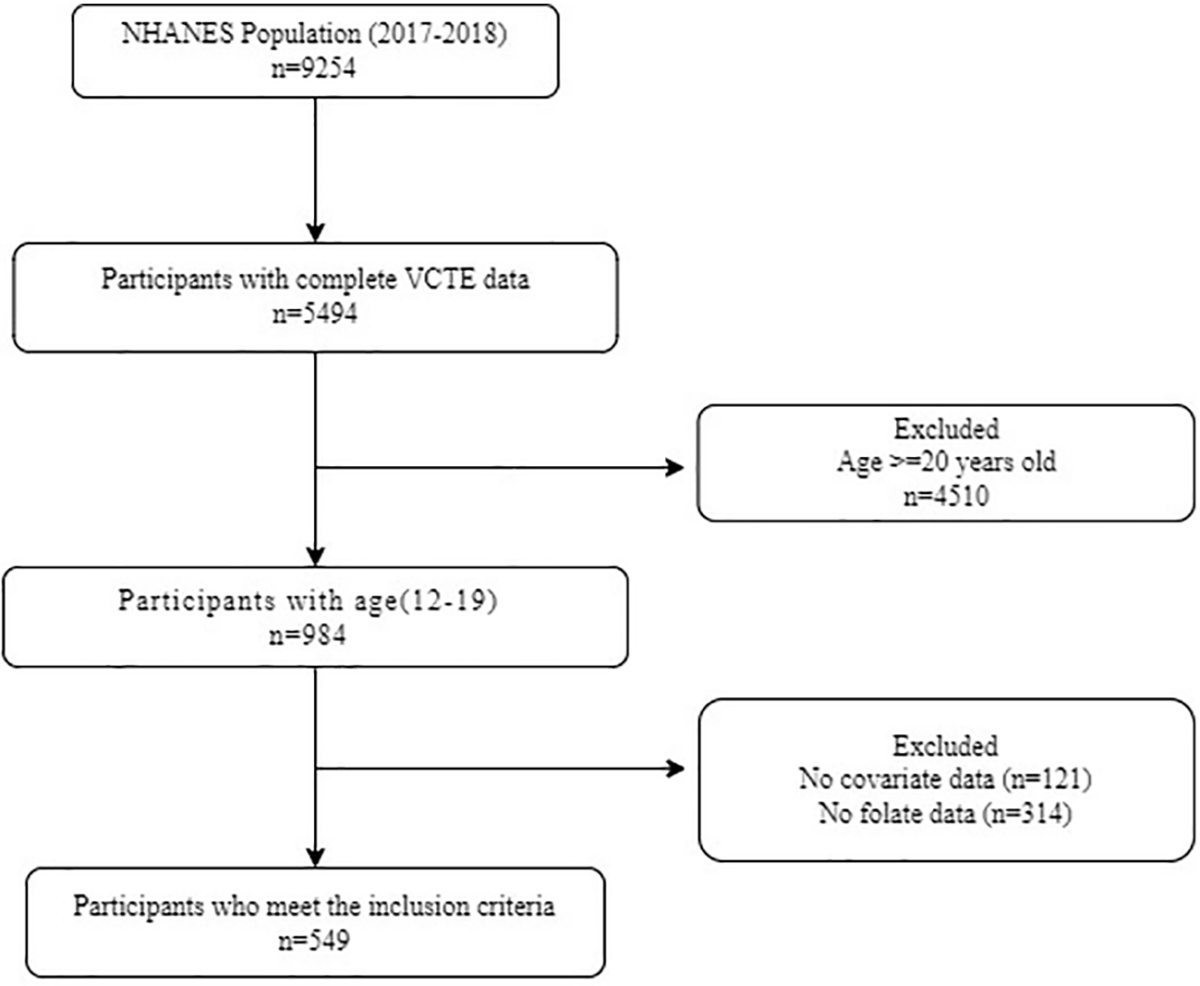
Flow diagram of study participants. NHANES, National Health and Nutrition Examination Survey; VCTE, vibration controlled transient elastography,.

The study analyzed the National Health and Nutrition Examination Survey (NHANES) data from the survey cycle of 2017 to 2018. The data was analyzed from December 2022 to May 2023. The NHANES is a nationally representative cross-sectional study. The NHANES has been approved by the Institutional Review Board of the National Institute for Nutrition and Health, and all data are accessible on https://www.cdc.gov/nchs/nhanes/index.htm. Among the 9254 participants in the 2017-2018 survey cycle, a total of 5494 participants have complete vibration-controlled transient elastography (VCTE) data. After excluding 4510 adults (≥ 20 years old), 314 participants with missing folate data and 121 participants with missing covariant data, the final cohort consists of 549 participants with complete data (**Figure 1**).

### Measurement of liver stiffness and hepatic steatosis

The NHANES examined all participants aged 12 and over with VCTE. If the participant (1) cannot lie on the examination table, (2) is pregnant, (3) has an implanted electronic medical device, (4) wears a bandage or a lesion near the ribs in the right abdomen or (5) refuses the examination or experiences a limited period of time during the examination, it is considered ineligible to undergo liver elastography. Participants were examined to evaluate CAP scores and liver stiffness measurements using the FibroScan model 502 V2 Touch equipped with a medium (M) or extra large (XL) wand (probe). If they have < 10 complete liver stiffness measurements or a liver stiffness interquartile (IQR) range/median ≥ 30%, or a fasting time < 3 h, transient elastography results are considered incomplete. The NHANES reported that the reliability of inter-observer CAP score was 0.94 and the reliability of liver stiffness test was 0.861 (19).

Firstly, the CAP and liver stiffness were analyzed as continuous variables. Then we defined NAFLD as the CAP score greater than or equal to 263dB/m (≥ S1) (20). Liver stiffness greater than or equal to 8kPa is considered to have significant fibrosis (≥F2) (21–23). We excluded participants with hepatitis B or hepatitis C and heavy drinkers(n=6) when defining NAFLD.

### Measurements of different folate forms

Serum and whole blood samples were collected by venipuncture and analyzed in the nutrition biomarker laboratory of the Centers for Disease Control and Prevention. In NHANES, five serum folate forms (5-methyl-tetrahydrofolate, folic acid, 5-formyl-tetrahydrofolate, tetrahydrofolate and 5,10-methenyl-tetrahydrofolate) were tested by liquid chromatography tandem mass spectrometry. The above five forms of folate were added together to calculate the total serum folate. The concentrations of serum total folate and whole blood folate were determined by microbiological method, and red blood cell folate was calculated. 5-methyl-tetrahydrofolate is the main bioactive form of serum total folate. The folic acid in our study refers to unmetabolized folic acid (UMFA) in the serum.The presence of UMFA in circulation may increase pro-inflammatory markers (24), reduce the cytotoxicity of natural killer cells, and damage DNA hydroxymethylation (25). Therefore, we speculate that the potential side effects of UMFA may mask the benefit of 5-methyltetrahydrofolic acid to NAFLD, and the relationship between serum folate and NAFLD may be different according to the form of serum folate. Therefore, we included red blood cell folate, serum total folate, 5-methyltetrahydrofolate and folic acid in the analysis. Except in restricted cubic splines, different forms of folate concentrations were naturally logtransformed to minimize the effect of outliers and to improve the interpretation of associational results.

### Covariates

Demographic covariates are obtained through self-reported questionnaires, including age, gender, race/ethnicity and income-to-poverty ratios. The income-to-poverty ratios is the ratio of household income to poverty. Trained health technicians measured the height and weight of the participants. Overweight is defined as BMI between the 85th and 95th percentile, and obesity is defined as BMI ≥ 95th percentile. Set the normal level as the reference level. In NHANES, participants’ smoking status (now, ever and never) was self-reported. The participants’ blood samples were sent to the NHANES laboratory for analysis to get the value of total cholesterol. Data on total dietary energy intake were obtained from a 24-hour dietary survey.

### Statistical analysis

The baseline feature is shown as mean ± SE of continuous variables or the percentage of classified variables. The baseline characteristics were compared according to the quartile of serum total folate, and the chi-square test of category variables or analysis of variance of continuous variables were used to analyze the differences.

We used linear regression models to evaluate the relationship between different folate forms and CAP or liver stiffness. The regression model is not adjusted at first, and then gradually adjusted according to the following factors: (1) age, sex, race/ethnicity and income-poverty ratio; (2) smoking status, total cholesterol and total energy intake; (3) BMI. Confounding factors were selected based on known CAP risk factors and folate-related risk factors in our data set. Then, we divided folate levels into four equal parts, and used the first quarter group as a reference category in the regression model to reanalyze the relationship between different folate forms and CAP or liver stiffness. We also used logistic regression to analyze the relationship between different folate forms and NAFLD or significant fibrosis including the above covariables in the adjusted models, and calculated the adjusted odds ratio (OR) and 95% confidence interval (CI).

To evaluate the potential nonlinear relationship between different folate forms and NAFLD or significant fibrosis, we performed a restricted cubic curve analysis (26). Three nodes located in the 5th, 50th and 95th percentiles of different folate forms are used in restricted cubic spline. Through the test of the spline, it is concluded whether there is a significant non-linear correlation.

We used regression-based intermediary analysis to distinguish between direct and BMI-mediated effects of folate on CAP or liver stiffness. Three estimates are as follows: (1) overall effects, that is, the overall association between folate and CAP or liver stiffness, including those mediated by BMI; (3) direct effects, that is, the association between folate and CAP or liver stiffness, adjusted according to BMI; and (3) indirect effects, that is, the association between folate and CAP or liver stiffness, mediated by BMI. In this study, the regression model of intermediary analysis is adjusted for all covariables.

We have done the sensitivity analysis in the following aspect. In order to test the independent correlation between 5-methyl-tetrahydrofolate or folic acid and NAFLD or significant fibrosis, we further mutually adjusted 5-methyl-tetrahydrofolate and folic acid. Use R (version 4.0.4) for analysis. The significance threshold was 0.05 and the bilateral P value was reported.

## Results

### Baseline characteristic of participants

A total of 549 participants were included in the final analysis, with an average age of 15.6 years old, of whom 34.4% were male. The means of CAP and liver stiffness were 223.02dB/m and 5.03kPa, respectively. The characteristics of participants classified by serum total folate quartile are shown in **Table 1**. We found that participants with higher serum total folate concentrations included younger, non-Hispanic white people, people who never smoked and had a low incidence of obesity. Similarly, we found that participants with higher serum total folate concentrations had lower CAP and liver stiffness (**Table 1**).

**Table 1:** Basic characteristics of the study participants.

Characteristics	Overall	Serum total folate, nmol/L				*P*
		Q1 [6.15, 27.70)	Q2 [27.70, 37.60)	Q3 [37.60, 50.70)	Q4 [50.70, 113.00]	
Participants	549	138	137	137	137	
Age, year						<0.001
Mean ± SE	15.6 ± 1.5	16.1 ± 1.4	15.9 ± 1.5	15.7 ± 1.5	14.7± 1.5	
Gender (%)						0.893
Male	189 (34.4)	47 (34.1)	48 (35.0)	50 (36.5)	44 (32.1)	
Female	360 (65.6)	91 (65.9)	89 (65.0)	87 (63.5)	93 (67.9)	
Race/ethnicity (%)						0.060
Non-Hispanic White	190 (34.6)	44 (31.9)	35 (25.5)	55 (40.1)	56 (40.9)	
Non-Hispanic Black	102 (18.6)	31 (22.5)	31 (22.6)	23 (16.8)	17 (12.4)	
Mexican American	115 (20.9)	30 (21.7)	30 (21.9)	25 (18.2)	30 (21.9)	
Other Hispanic	33 (6.0)	7 (5.1)	11 (8.0)	3 (2.2)	12 (8.8)	
Other Race	109 (19.9)	26 (18.8)	30 (21.9)	31 (22.6)	22 (16.1)	
Income-poverty ratio (%)						0.280
≤ 2	322 (58.7)	82 (59.4)	83 (60.6)	71 (51.8)	86 (62.8)	
> 2	227 (41.3)	56 (40.6)	54 (39.4)	66 (48.2)	51 (37.2)	
Total cholesterol, mg/dL						0.268
Mean ± SE	156 ± 5	154 ± 6	153 ± 5	160 ± 5	156 ± 6	
Total energy, kcal/day						0.479
Mean ± SE	1846 ± 27	1828 ± 27	1838 ± 27	1821 ± 27	1898 ± 26	
Smoking (%)						0.040
never	535 (97.4)	129 (93.5)	134 (97.8)	136(99.3)	136 (99.3)	
ever	5 (0.9)	3 (2.2)	1 (0.7)	1 (0.7)	0 (0)	
now	9 (1.6)	6 (4.3)	2 (1.5)	0 (0)	1 (0.7)	
BMI (%)						0.005
normal	310 (56.6)	72 (52.2)	77 (56.2)	67 (48.9)	94 (68.6)	
overweight	96 (17.5)	24 (17.4)	18 (13.1)	31 (22.6)	23 (16.8)	
obesity	143 (26.0)	42 (30.4)	42 (30.7)	39 (28.5)	20 (14.6)	
CAP, dB/m						0.002
Mean ± SE	223.02 ± 7.50	229.59 ± 7.75	227.88 ± 7.35	225.91 ± 7.35	208.66 ± 7.41	
Liver Stiffness, kPa						0.006
Mean ± SE	5.03 ± 1.83	5.66 ± 2.42	5.13 ± 1.58	4.71 ± 1.08	4.62 ± 1.26	

BMI, body mass index; CAP, Controlled Attenuation Parameter.

### Relationship between different folate forms and CAP

We first analyzed the relationship between different folate forms and CAP. In model 2, there was a negative correlation between serum total folate (β: -18.53; 95%CI: -29.32 to -7.73) or 5-methyltetrahydrofolate (β: -14.13; 95%CI: -28.98 to -7.86) and CAP. However, when the BMI was further adjusted in model 3, this negative correlation no longer existed (serum total folate: β: -8.36; 95%CI: -17.69 to 0.97; 5-methyltetrahydrofolate: β: -8.05; 95%CI: -17.19 to 1.09). There was no significant correlation between red blood cell folate or folic acid and CAP in either model 2 or model 3. After changing folate from a continuous variable to a classified variable (quartile), we get the same results (**Table 2**).

**Table 2:** Associations of different folate forms with CAP among teenagers aged 12-19 years(n=549).

Folate	Unadjusted model			Adjusted model								
				Model 1 ^a^			Model 2 ^b^			Model 3 ^c^		
	β	CI (95%)	*P*	β	CI (95%)	*P*	β	CI (95%)	*P*	β	CI (95%)	*P*
Red blood cell folate (continuous), nmol/L	3.75	-11.31 to 18.82	0.626	2.61	-12.49 to 17.71	0.735	1.81	-13.30 to 16.91	0.815	3.28	-9.53 to 16.09	0.616
Red blood cell folate (categorical), nmol/L												
Q1	Ref			Ref			Ref			Ref		
Q2	0.29	-12.98 to 13.56	0.966	-1.46	-14.54 to 11.63	0.828	-2.50	-15.66 to 10.66	0.710	-2.03	-13.20 to 9.13	0.721
Q3	13.73	0.47 to 27.01	0.043	9.57	-3.70 to 22.85	0.158	8.44	-4.86 to 21.73	0.214	7.91	-3.36 to 19.19	0.169
Q4	2.26	-11.01 to 15.53	0.738	1.23	-12.00 to 14.46	0.855	-0.11	-13.37 to 13.15	0.987	0.94	-10.31 to 12.20	0.870
P for trend	0.346			0.507			0.639			0.498		
Serum total folate (continuous), nmol/L	-18.81	-29.29 to -8.33	<.001	-16.94	-27.64 to -6.24	0.002	-18.53	-29.32 to -7.73	0.001	-8.36	-17.69 to 0.97	0.080
Serum total folate (categorical), nmol/L												
Q1	Ref			Ref			Ref			Ref		
Q2	-1.72	-14.90 to 11.47	0.799	-1.97	-14.93 to 10.98	0.765	-2.63	-15.59 to 10.34	0.691	-1.01	-12.12 to 10.09	0.858
Q3	-3.68	-16.87 to 9.51	0.584	-1.99	-15.01 to 11.03	0.765	-4.09	-17.20 to 9.02	0.541	-2.97	-14.20 to 8.27	0.605
Q4	-20.94	-34.12 to -7.75	0.002	-19.85	-33.23 to -6.47	0.004	-21.09	-34.50 to -7.68	0.002	-7.48	-19.12 to 4.16	0.208
P for trend	0.003			0.007			0.003			0.199		
5-Methyl-tetrahydrofolate (continuous), nmol/L	-18.56	-28.80 to -8.32	<.001	-13.69	-27.26 to -6.34	0.002	-14.13	-28.98 to -7.86	0.001	-8.05	-17.19 to 1.09	0.085
5-Methyl-tetrahydrofolate (categorical), nmol/L												
Q1	Ref			Ref			Ref			Ref		
Q2	-1.71	-14.91 to 11.48	0.799	-1.69	-14.63 to 11.26	0.798	-2.07	-15.03 to 10.90	0.755	-1.27	-12.38 to 9.83	0.822
Q3	-2.43	-15.62 to 10.76	0.718	-1.29	-14.34 to 11.75	0.846	-3.49	-16.64 to 9.66	0.603	-2.54	-13.79 to 8.72	0.659
Q4	-20.28	-33.47 to -7.08	0.003	-19.34	-32.70 to -5.99	0.005	-20.19	-33.57 to -6.80	0.003	-7.30	-18.89 to 4.29	0.218
P for trend	0.004			0.009			0.005			0.222		
Folic acid (continuous), nmol/L	-2.44	-10.48 to 5.61	0.553	0.298	-7.71 to 8.30	0.942	0.745	-7.26 to 8.75	0.855	1.44	-5.36 to 8.23	0.679
Folic acid (categorical), nmol/L												
Q1	Ref			Ref			Ref			Ref		
Q2	-1.39	-14.71 to 11.93	0.838	-0.216	-13.38 to 12.95	0.974	0.18	-12.97 to 13.34	0.978	-1.19	-12.34 to 9.96	0.834
Q3	-5.36	-18.68 to 7.96	0.430	-1.82	-15.08 to 11.43	0.788	-1.11	-14.36 to 12.14	0.870	-3.49	-14.73 to 7.75	0.543
Q4	-6.87	-20.19 to 6.45	0.313	-2.55	-15.84 to 10.75	0.707	-1.74	-15.06 to 11.58	0.798	2.43	-8.87 to 13.73	0.674
P for trend	0.253			0.668			0.762			0.789		

BMI, body mass index; CAP, Controlled Attenuation Parameter; CI, confidence interval.

a Adjusted for gender, age, race/ethnicity, income-poverty ratio.

b Adjusted for gender, age, race/ethnicity, income-poverty ratio, total energy, total cholesterol, smoking status.

c Adjusted for gender, age, race/ethnicity, income-poverty ratio, total energy, total cholesterol, smoking status and BMI.

We also discussed the relationship between folate and the prevalence of NAFLD in adolescents. In model 2, higher serum total folate (OR: 0.49; 95%CI: 0.29 to 0.81) or 5-methyltetrahydrofolate (OR: 0.48; 95%CI: 0.29 to 0.78) was associated with less NAFLD disease, regardless of whether serum total folate was a continuous variable or a classification variable (quartile). After adding BMI to the model, this correlation disappeared (serum total folate: OR: 0.71; 95%CI: 0.38 to 1.31; 5-methyltetrahydrofolate: OR: 0.69; 95%CI: 0.37 to 1.26). Similarly, we did not find this relationship in the analysis of red blood cell folate and folic acid (**eTable 1**).

We also used restricted cubic splines to analyze the nonlinear relationship between different folate forms and the prevalence of NAFLD, but did not find a significant nonlinear correlation between them (**eFigures 1–4**).

### Relationship between different folate forms and liver stiffness

Except for the edge significance of model 2, the relationship between different folate forms and liver stiffness was similar to that between different folate forms and CAP (**Table 3**). Similarly, we found a negative correlation between serum total folate or 5-Methyl-tetrahydrofolate and significant fibrosis (**eTable 2**). The nonlinear relationship between different folate forms and significant fibrosis was not significant (**eFigures 5–8**).

**Table 3:** Associations of different folate forms with liver stiffness among teenagers aged 12-19 years (n=549).

Folate	Unadjusted model			Adjusted model								
				Model 1 ^a^			Model 2 ^b^			Model 3 ^c^		
	β	CI (95%)	*P*	β	CI (95%)	*P*	β	CI (95%)	*P*	β	CI (95%)	*P*
Red blood cell folate (continuous), nmol/L	-0.67	-1.57 to 0.23	0.144	-0.59	-1.51 to 0.32	0.203	-0.34	-1.22 to 0.54	0.455	-0.31	-1.18 to 0.57	0.492
Red blood cell folate (categorical), nmol/L												
Q1	Ref			Ref			Ref			Ref		
Q2	-0.46	-1.25 to 0.34	0.259	-0.45	-1.24 to 0.34	0.264	-0.41	-1.18 to 0.36	0.296	-0.40	-1.16 to 0.37	0.310
Q3	-0.15	-0.95 to 0.64	0.706	-0.07	-0.88 to 0.73	0.858	0.15	-0.63 to 0.92	0.712	0.14	-0.63 to 0.91	0.719
Q4	-0.53	-1.32 to 0.27	0.196	-0.44	-1.25 to 0.36	0.280	-0.27	-1.05 to 0.50	0.487	-0.25	-1.01 to 0.52	0.528
P for trend	0.322			0.457			0.818			0.858		
Serum total folate (continuous), nmol/L	-1.13	-1.76 to -0.51	<.001	-1.06	-1.71 to -0.41	0.001	-0.72	-1.35 to -0.09	0.026	-0.57	-1.21 to 0.07	0.079
Serum total folate (categorical), nmol/L												
Q1	Ref			Ref			Ref			Ref		
Q2	-0.53	-1.32 to 0.27	0.193	-0.57	-1.36 to 0.22	0.160	-0.37	-1.14 to 0.37	0.335	-0.36	-1.12 to 0.40	0.351
Q3	-0.95	-1.74 to -0.16	0.019	-0.87	-1.66 to -0.07	0.033	-0.53	-1.30 to 0.24	0.177	-0.50	-1.27 to 0.26	0.197
Q4	-1.04	-1.83 to -0.25	0.010	-0.94	-1.76 to -0.13	0.024	-0.69	-1.48 to 0.10	0.087	-0.49	-1.28 to 0.31	0.230
P for trend	0.006			0.017			0.079			0.201		
5-Methyl-tetrahydrofolate (continuous), nmol/L	-1.14	-1.75 to -0.53	<.001	-1.07	-1.70 to -0.44	0.001	-0.73	-1.35 to -0.11	0.021	-0.58	-1.20 to 0.04	0.068
5-Methyl-tetrahydrofolate (categorical), nmol/L												
Q1	Ref			Ref			Ref			Ref		
Q2	-0.47	-1.26 to 0.32	0.248	-0.50	-1.29 to 0.29	0.216	-0.33	-1.09 to 0.43	0.394	-0.33	-1.09 to 0.42	0.389
Q3	-0.97	-1.76 to -0.18	0.017	-0.89	-1.68 to -0.09	0.029	-0.56	-1.33 to 0.21	0.154	-0.54	-1.31 to 0.23	0.168
Q4	-1.02	-1.81 to -0.23	0.012	-0.91	-1.72 to -0.09	0.029	-0.67	-1.45 to 0.12	0.097	-0.47	-1.26 to 0.32	0.241
P for trend	0.005			0.017			0.078			0.194		
Folic acid (continuous), nmol/L	-0.10	-0.59 to 0.38	0.673	-0.06	-0.55 to 0.42	0.799	-0.06	-0.52 to 0.41	0.816	-0.05	-0.51 to 0.41	0.828
Folic acid (categorical), nmol/L												
Q1	Ref			Ref			Ref			Ref		
Q2	0.80	0.01 to 1.59	0.049	0.76	-0.04 to 1.55	0.063	0.76	-0.01 to 1.52	0.052	0.73	-0.03 to 1.49	0.059
Q3	0.16	-0.63 to 0.95	0.686	0.18	-0.62 to 0.98	0.654	0.20	-0.56 to 0.98	0.596	0.18	-0.58 to 0.94	0.644
Q4	0.05	-0.74 to 0.84	0.901	0.14	-0.67 to 0.94	0.739	0.14	-0.63 to 0.91	0.722	0.20	-0.57 to 0.97	0.610
P for trend	0.710			0.915			0.927			0.960		

BMI, body mass index; CI, confidence interval; OR, Odds Ratio.

a Adjusted for gender, age, race/ethnicity, income-poverty ratio.

b Adjusted for gender, age, race/ethnicity, income-poverty ratio, total energy, total cholesterol, smoking status.

c Adjusted for gender, age, race/ethnicity, income-poverty ratio, total energy, total cholesterol, smoking status and BMI.

### Mediating role of BMI

Because the adjustment of BMI in model 3 masked the correlation between serum total folate or 5-methyl-tetrahydrofolate and CAP or liver stiffness, we further analyzed the mediating effect of BMI. All mediating analyses are adjusted based on gender, age, race/ethnicity, income-poverty ratio, total energy, total cholesterol and smoking status. It is estimated that 76% of the total association between serum total folate and CAP is mediated by BMI. BMI also significantly regulated the relationship between serum total folate and liver stiffness. The mediating proportion of BMI in the total correlation between serum total folate and liver stiffness was 50%. Similarly, we found that BMI significantly mediated the relationship between 5-methyl-tetrahydrofolate and CAP or liver stiffness, with a mediating ratio of 77% and 49%, respectively (**Tables 4**, **5**).

**Table 4:** Estimated proportion of different folate forms with CAP mediated by BMI.

Measure	Red blood cell folate, nmol/L		Serum total folate, nmol/L		5-Methyl-tetrahydrofolate, nmol/L		Folic acid, nmol/L	
	β (95% CI)	*P* Value	β (95% CI)	*P* Value	β (95% CI)	*P* Value	β (95% CI)	*P* Value
Exposure to mediator	-0.15 (-1.96 to 1.66)	0.874	-2.86 (-4.14 to -1.58)	<.001	-2.87 (-4.13 to -1.63)	<.001	-0.28 (-0.29 to 0.68)	0.569
Mediator to outcome	4.76 (4.19 to 5.34)	<.001	4.76 (4.19 to 5.34)	<.001	4.76 (4.19 to 5.34)	<.001	4.76 (4.19 to 5.34)	<.001
Direct effect	2.83 (-10.22 to 15.42)	0.647	-4.23 (-13.89 to 5.84)	0.412	-4.06 (-13.53 to 5.96)	0.416	2.09 (-3.29 to 7.91)	0.449
Indirect effect	-0.70 (-10.45 to 8.35)	0.882	-13.50 (-20.27 to -6.96)	<.001	-13.57 (-20.17 to -7.14)	<.001	-1.34 (-5.58 to 2.64)	0.553
Total effect	2.13 (-12.30 to 17.30)	0.773	-17.74 (-29.62 to -6.65)	0.002	-17.64 (-29.16 to -6.75)	0.002	0.75 (-6.37 to 7.87)	0.794
Proportion mediated, %	NA		76	0.002	77	0.002	NA	

BMI, body mass index; CI, confidence interval; CAP, Controlled Attenuation Parameter.

Adjusted for Adjusted for gender, age, race/ethnicity, income-poverty ratio, total energy, total cholesterol and smoking status.

**Table 5:** Estimated proportion of different folate forms with liver stiffness mediated by BMI.

Measure	Red blood cell folate, nmol/L		Serum total folate, nmol/L		5-Methyl-tetrahydrofolate, nmol/L		Folic acid, nmol/L	
	β (95% CI)	*P* Value	β (95% CI)	*P* Value	β (95% CI)	*P* Value	β (95% CI)	*P* Value
Exposure to mediator	-0.15 (-1.96 to 1.66)	0.874	-2.86 (-4.14 to -1.58)	<.001	-2.87 (-4.13 to -1.63)	<.001	-0.28 (-0.29 to 0.68)	0.569
Mediator to outcome	0.17 (0.13 to 0.21)	<.001	0.17 (0.13 to 0.21)	<.001	0.17 (0.13 to 0.21)	<.001	0.17 (0.13 to 0.21)	<.001
Direct effect	-0.51 (-1.83 to 0.42)	0.482	-0.48 (-1.08 to -0.06)	0.020	-0.50 (-1.08 to -0.08)	0.014	0.02 (-0.25 to 0.31)	0.890
Indirect effect	-0.03 (-0.68 to 0.27)	0.881	-0.47 (-1.53 to -0.05)	<.001	-0.48 (-1.54 to -0.05)	<.001	-0.04 (-0.30 to 0.12)	0.834
Total effect	-0.53 (-2.22 to 0.46)	0.478	-0.97 (-2.33 to -0.23)	0.004	-0.98 (-2.35 to -0.24)	0.004	-0.03 (-0.25 to 0.22)	0.827
Proportion mediated, %	NA		50	0.004	49	0.004	NA	

BMI, body mass index; CI, confidence interval.

Adjusted for Adjusted for gender, age, race/ethnicity, income-poverty ratio, total energy, total cholesterol and smoking status.

### Sensitivity analyses

After the mutual adjustment of 5-methyl-tetrahydrofolate and folic acid, the negative correlation between 5-methyl-tetrahydrofolate and CAP was still significant, but the negative correlation between 5-methyl-tetrahydrofolate and liver stiffness was weakened. After further adjustment, there was still no significant correlation between folic acid and CAP or liver stiffness (**eTable 3**).

## Discussion

Our data showed that without further adjustment for BMI in the model, serum total folate and 5-methyl-tetrahydrofolate were independently associated with CAP and liver stiffness, regardless of whether folate form was a continuous or categorical variable. Serum total folate and 5-methyl-tetrahydrofolate were also independently associated with NAFLD and significant fibrosis. However, after further adjustment for BMI, this significance disappeared. We also found that there was no significant correlation between red blood cell folate or folic acid and CAP or liver stiffness, regardless of BMI adjustment.The nonlinear relationships between different forms of folate and NAFLD and significant fibrosis were also not significant. The results of sensitivity analysis also support these conclusions. In mediation analysis, we found that BMI fully mediated the correlation between serum total folate or 5-methyl-tetrahydrofolate and CAP, and partially mediated the correlation between serum total folate or 5-methyl-tetrahydrofolate and liver stiffness.

Numerous studies have shown that serum folate levels are associated with liver steatosis and liver stiffness in adults (13–15, 27), but to date, to our knowledge, no studies have explored the relationship between serum folate and liver steatosis or liver stiffness in adolescents. Our study fills this gap. Folate may also serve as a biomarker or potential treatment for hepatic steatosis and liver stiffness, not only in adults but also in adolescents.

One study conducted in adults showed that red blood cell folate was independently associated with an increased risk of NAFLD (28). Another study showed that higher UMFA concentrations in adults were significantly associated with a higher prevalence of NAFLD (15). We found no such correlation in our study, and we speculate that there are two reasons for this. First, the age of the population we studied was different. Previous studies have linked red blood cell folate to insulin resistance and metabolic syndrome (29). However, insulin resistance and metabolic syndrome are uncommon in adolescents. Also because of younger age, folic acid accumulation is less. Therefore, in our study, red blood cell folate and folic acid were not found to be associated with the prevalence of NAFLD. Second, we define nonalcoholic fatty liver disease differently. The above studies mainly defined NAFLD by the United States fatty liver index or the fatty liver index, whereas our study defined NAFLD by CAP of transient elastography.

The underlying mechanism by which serum folate levels are negatively correlated with hepatic steatosis and liver stiffness can be explained in different ways. First, the risk of hepatic steatosis is elevated in the context of folate deficiency, possibly because folate deficiency is associated with increased expression of lipid biosynthesis genes, resulting in disruption of liver lipid metabolism (30). Secondly, some studies have shown that lipid transport is blocked in the liver of folate-deficient animals, thus promoting liver fat accumulation (31–33). Furthermore, the deficiency of one carbon unit of folate binding interferes with purine signaling and accelerates the progression of liver fibrosis (34). In addition, folate regulates microRNA expression in the liver, reduces blood glucose and lipid concentrations, increases insulin sensitivity, and improves liver function (35). Finally, folate deficiency interferes with the fibroblast growth factor path (36).

Folate has a powerful antioxidant function for human health and is able to directly remove reactive oxygen species (37). Oxidative stress may be involved in the pathogenesis of NAFLD by promoting inflammation (38, 39). In addition, several clinical studies have shown that reduced serum folate levels are associated with increased BMI and are associated with insulin resistance (24, 40), which is considered a risk factor for NAFLD. Some studies have also shown that a higher BMI predicts more inflammatory cytokines (41). The above may be the reason why BMI plays a major mediating role in the negative correlation between serum folate concentration and hepatic steatosis and liver stiffness.

Our research has the following advantages. First of all, the population we studied came from a nationally representative cross-sectional survey. Secondly, we use transient elastography to define NAFLD and measure liver stiffness. As a simple, non-invasive and accurate technique, transient elastography is considered as a non-invasive standard tool for the evaluation of liver fibrosis. Transient elastography also included a control attenuation parameter (CAP) score, which measures ultrasonic attenuation associated with hepatic steatosis. Third, we use multiple models and multiple dimensions (continuous variables and classified variables, linear and nonlinear relations and various sensitivity analysis) to prove our conclusion.

However, this study also has some limitations. First of all, due to the limitations of the cross-sectional design of the study, the temporal causality may not be cautious. Therefore, reverse causality and unmeasured residue confusion may prevent causal inferences from the association between different forms of folate levels and hepatic steatosis and liver stiffness. A prospective study is needed to confirm or refute our observations. Second, there are currently no general cutoff guidelines for CAP score and liver stiffness among adolescents. However, the cutoff points for CAP score and liver stiffness we used came from several good studies (3–5). Finally, because there are fewer smokers among teenagers, as a covariable, this will affect our results to some extent. These differences may lead to selection bias. Therefore, our results are carefully interpreted.

## Conclusion

Our results show that serum total folate or 5-Methyl-tetrahydrofolate are negatively correlated with hepatic steatosis or liver stiffness in adolescents, and BMI plays major mediating role in this relationship. Our findings emphasize the importance of monitoring the concentration of serum folate, not just the serum total folate concentration. The results of this study can provide guidance for the biomarker effect of serum folate level and drug therapy, so as to prevent hepatic steatosis and liver sclerosis more effectively. Since this study is the first attempt to investigate the relationship between different folate forms and hepatic steatosis or liver stiffness in adolescents, more longitudinal and intervention studies are needed in the future.

## Data availability statement

Data described in the article and code book are publicly accessible online via the NHANES website (https://wwwn.cdc.gov/nchs/nhanes/). Analytic code will be made available upon request.

## Ethics statement

The studies involving humans were approved by the Institutional Review Board of the National Institute for Nutrition and Health. The studies were conducted in accordance with the local legislation and institutional requirements. Written informed consent for participation in this study was provided by the participants’ legal guardians/next of kin.

## Author contributions

JW: Conceptualization, Data curation, Formal analysis, Investigation, Methodology, Project administration, Resources, Software, Supervision, Validation, Visualization, Writing – original draft, Writing – review & editing. YF: Data curation, Investigation, Methodology, Writing – original draft. LY: Data curation, Investigation, Methodology, Writing – original draft. KL: Data curation, Investigation, Methodology, Software, Writing – original draft. QX: Data curation, Investigation, Methodology, Writing – original draft. XC: Data curation, Investigation, Methodology, Writing – original draft. JS: Data curation, Investigation, Methodology, Writing – original draft. YZ: Data curation, Investigation, Methodology, Writing – original draft. ZZ: Conceptualization, Data curation, Funding acquisition, Investigation, Methodology, Project administration, Resources, Software, Supervision, Writing – original draft, Writing – review & editing.
