# Supplementary material for: Analysis of the mediating role of BMI in associations of different folate forms with hepatic steatosis and liver fibrosis in adolescents in the USA: results from the NHANES 2017-2018
Source: Front Endocrinol (Lausanne). 2023 Dec 5;14:1273580. doi: 10.3389/fendo.2023.1273580 (PMC10728716; doi:10.3389/fendo.2023.1273580)
Supplement: Supplementary file 1 [file DataSheet_1.docx]

**Supplemental Online Content**

**eFigure 1.** Nonlinear association between red blood cell folate and NAFLD.

**eFigure 2.** Nonlinear association between serum total folate and NAFLD.

**eFigure 3.** Nonlinear association between 5-methyl-tetrahydrofolate and NAFLD.

**eFigure 4.** Nonlinear association between folic acid and NAFLD.

**eFigure 5.** Nonlinear association between red blood cell folate and significant fibrosis.

**eFigure 6.** Nonlinear association between serum total folate and significant fibrosis.

**eFigure 7.** Nonlinear association between 5-methyl-tetrahydrofolate and significant fibrosis.

**eFigure 8.** Nonlinear association between folic acid and significant fibrosis.

**eTable 1.** Associations of different folate forms with NAFLD among teenagers aged 12-19

years(excluding participants with significant alcohol consumption or viral hepatitis)(n=543).

**eTable 2.** Associations of different folate forms with significant fibrosis(>8 kPa) among teenagers

aged 12-19 years(n=549).

**eTable 3.** Associations of different folate forms with CAP and liver stiffness among teenagers aged12-19 years(mutually adjustment for 5-methyl-tetrahydrofolate and folic acid)(n=549).

**
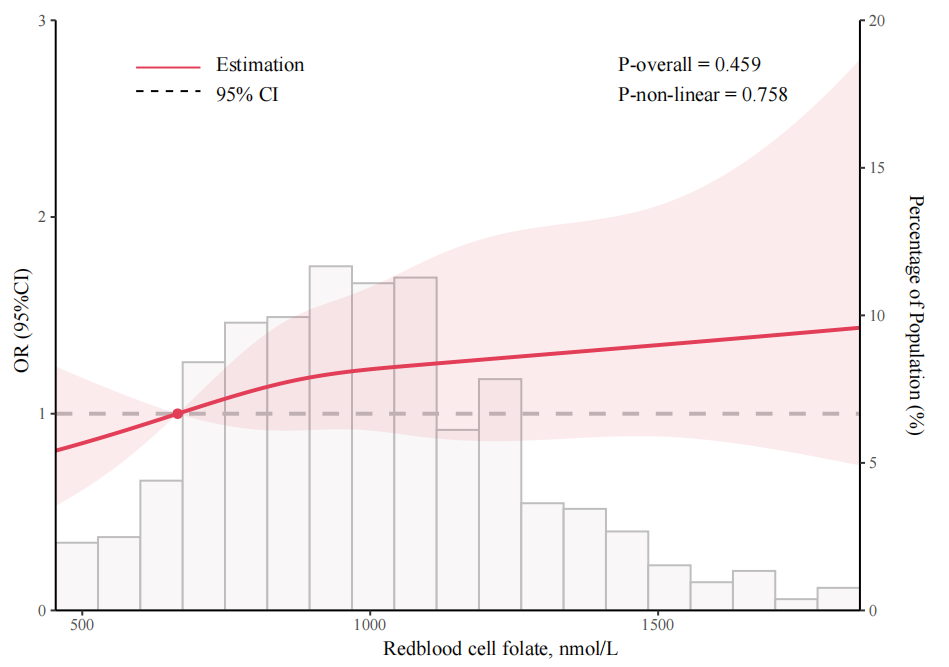
**

**eFigure 1.** Nonlinear association between red blood cell folate and NAFLD.


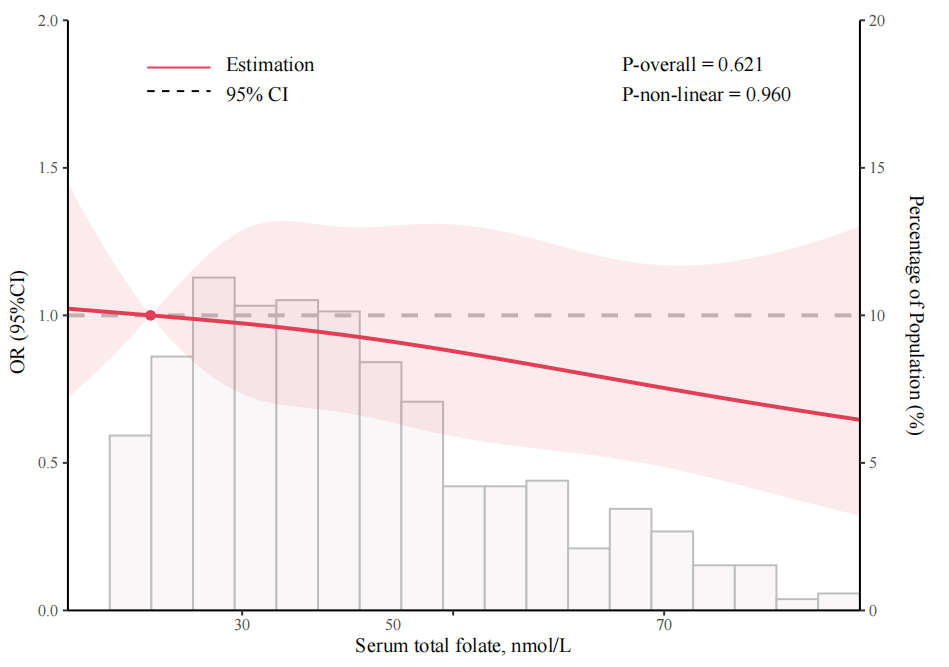


**eFigure 2.** Nonlinear association between serum total folate and NAFLD.


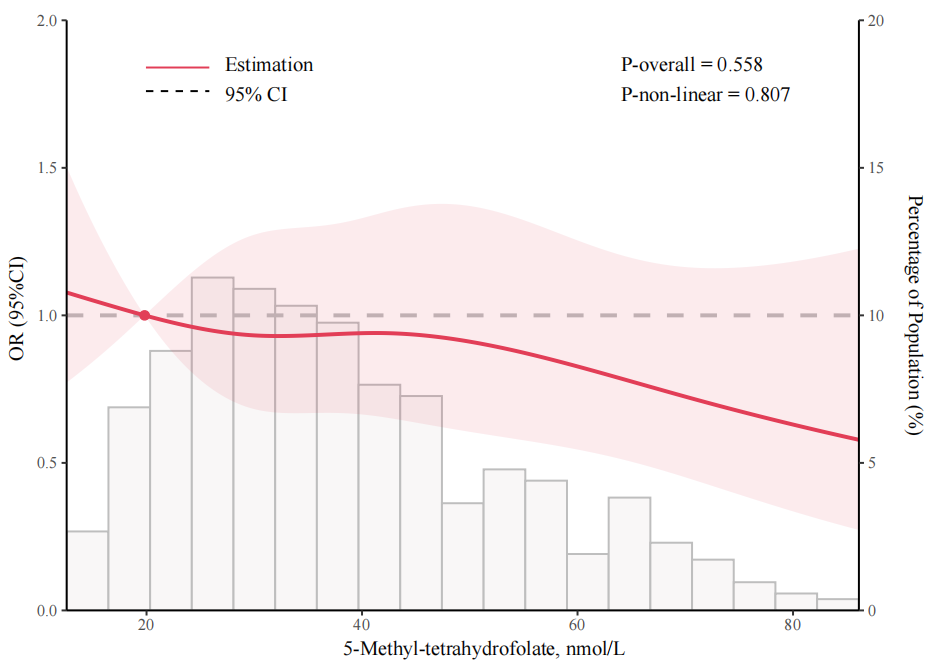


**eFigure 3.** Nonlinear association between 5-methyl-tetrahydrofolate and NAFLD.


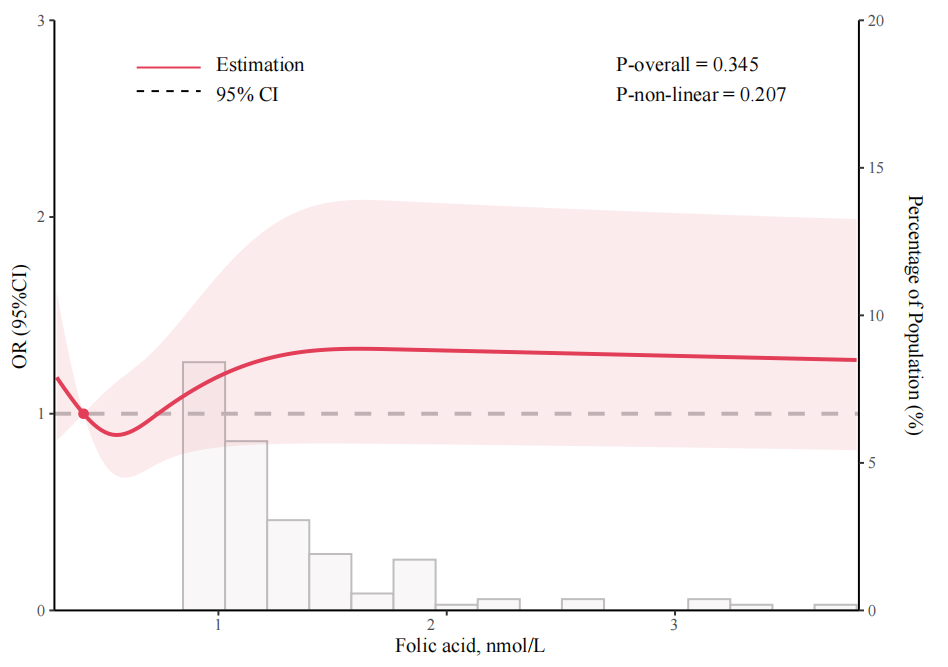


**eFigure 4.** Nonlinear association between folic acid and NAFLD.


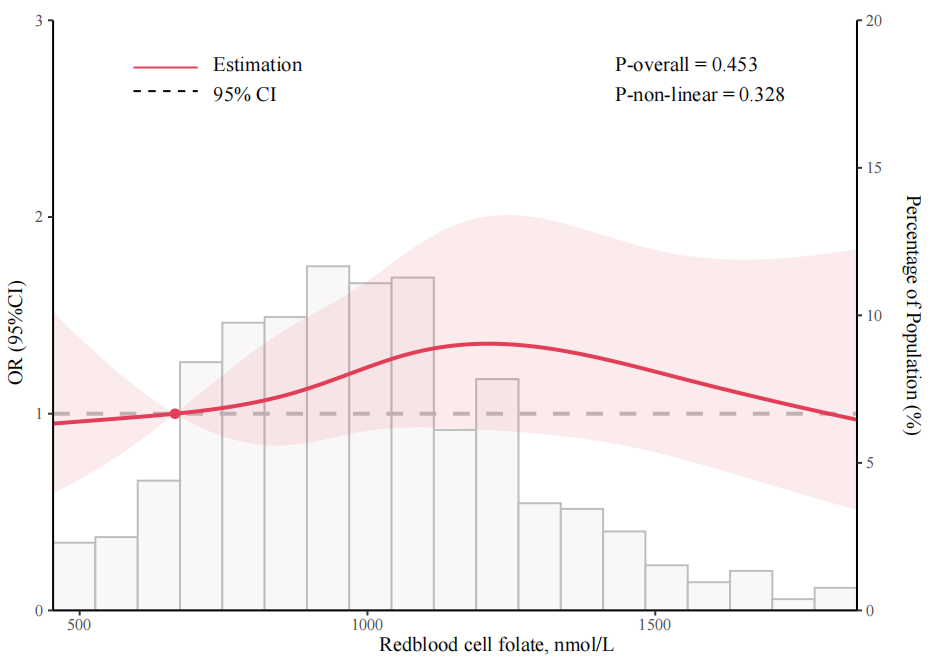


**eFigure 5.** Nonlinear association between red blood cell folate and significant fibrosis.


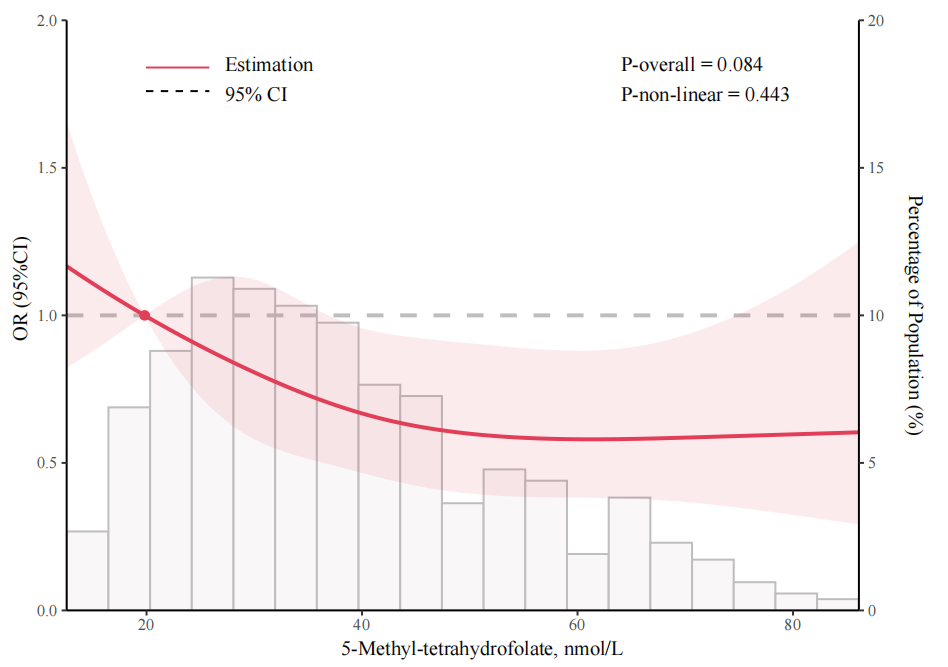


**eFigure 6.** Nonlinear association between serum total folate and significant fibrosis.


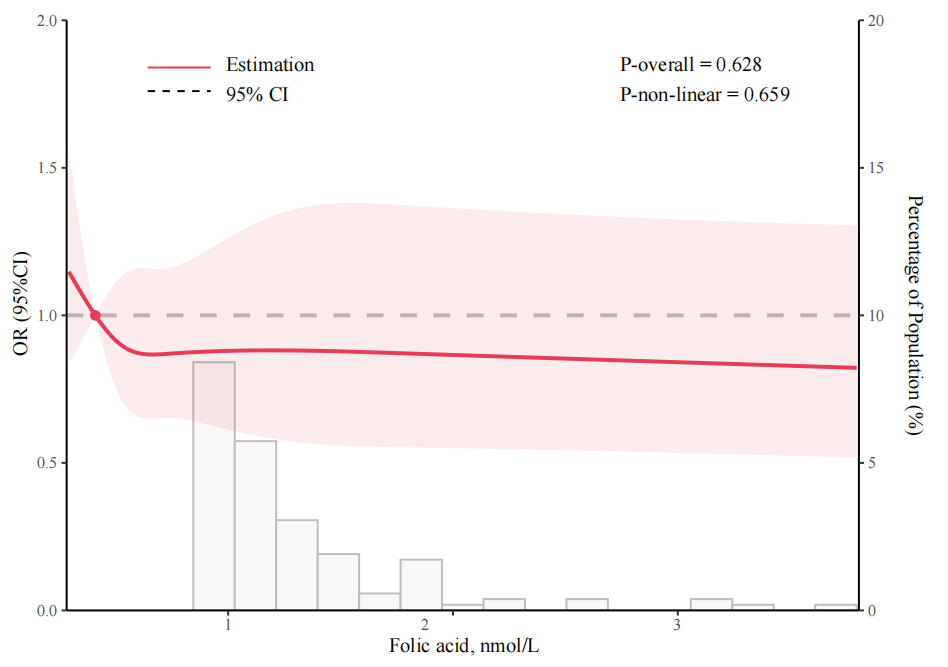


**eFigure 7.** Nonlinear association between 5-methyl-tetrahydrofolate and significant fibrosis.


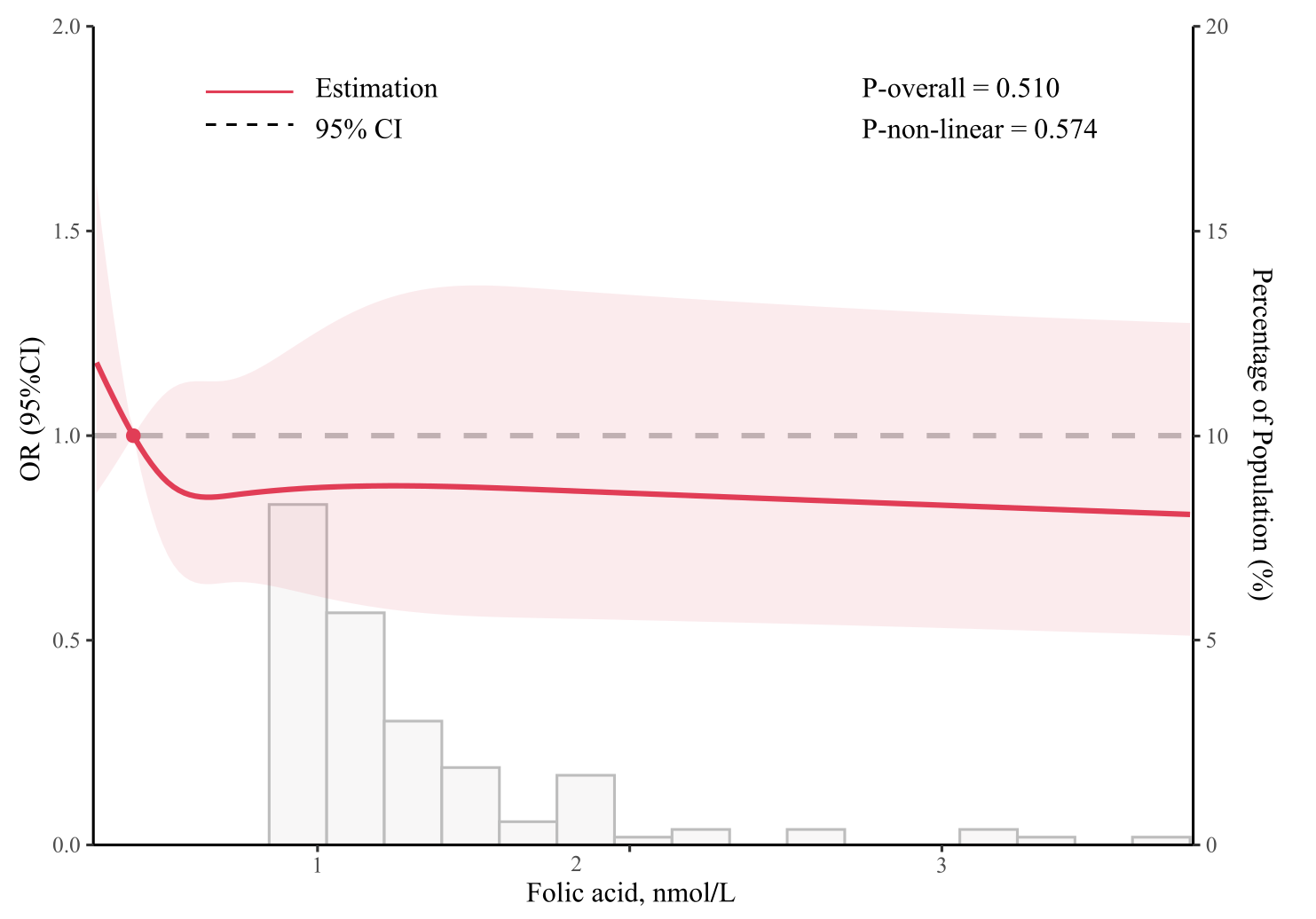


**eFigure 8.** Nonlinear association between folic acid and significant fibrosis.

**eTable 1**. Associations of different folate forms with NAFLD among teenagers aged 12-19 years(excluding participants with significant alcohol consumption or viral hepatitis)(n=543).

|  |  | | | **Adjusted model** | | | | | | | | |
| --- | --- | --- | --- | --- | --- | --- | --- | --- | --- | --- | --- | --- |
| **Folate** | **Unadjusted model** | | | **Model 1 ^a^** | | | **Model 2 ^b^** | | | **Model 3 ^c^** | | |
|  | **OR** | **CI (95%)** | ***P*** | **OR** | **CI (95%)** | ***P*** | **OR** | **CI (95%)** | ***P*** | **OR** | **CI (95%)** | ***P*** |
| Red blood cell folate (continuous), nmol/L | 1.23 | 0.63 to 2.43 | 0.547 | 1.16 | 0.57 to 2.35 | 0.688 | 1.09 | 0.53 to 2.27 | 0.810 | 1.28 | 0.51 to 3.24 | 0.603 |
| Red blood cell folate (categorical), nmol/L |  |  |  |  |  |  |  |  |  |  |  |  |
| Q1 | Ref |  |  | Ref |  |  | Ref |  |  | Ref |  |  |
| Q2 | 0.95 | 0.50 to 1.78 | 0.873 | 0.90 | 0.47 to 1.70 | 0.734 | 0.82 | 0.42 to 1.58 | 0.554 | 0.77 | 0.34 to 1.69 | 0.508 |
| Q3 | 1.68 | 0.94 to 3.04 | 0.081 | 1.50 | 0.82 to 2.75 | 0.190 | 1.40 | 0.76 to 2.60 | 0.284 | 1.67 | 0.79 to 3.57 | 0.183 |
| Q4 | 1.22 | 0.67 to 2.25 | 0.518 | 1.17 | 0.60 to 2.06 | 0.629 | 1.06 | 0.56 to 2.02 | 0.849 | 1.21 | 0.56 to 2.63 | 0.634 |
| P for trend | 0.308 | | | 0.445 | | | 0.646 | | | 0.291 | | |
| Serum total folate (continuous), nmol/L | 0.55 | 0.34 to 0.88 | 0.013 | 0.56 | 0.34 to 0.91 | 0.019 | 0.49 | 0.29 to 0.81 | 0.006 | 0.71 | 0.38 to 1.31 | 0.271 |
| Serum total folate (categorical), nmol/L |  |  |  |  |  |  |  |  |  |  |  |  |
| Q1 | Ref |  |  | Ref |  |  | Ref |  |  | Ref |  |  |
| Q2 | 0.96 | 0.55 to 1.68 | 0.887 | 0.94 | 0.53 to 1.66 | 0.823 | 0.92 | 0.51 to 1.64 | 0.774 | 1.01 | 0.50 to 2.04 | 0.987 |
| Q3 | 0.85 | 0.48 to 1.49 | 0.563 | 0.88 | 0.49 to 1.58 | 0.677 | 0.77 | 0.43 to 1.40 | 0.399 | 0.77 | 0.37 to 1.58 | 0.476 |
| Q4 | 0.45 | 0.23 to 0.84 | 0.015 | 0.43 | 0.21 to 0.82 | 0.013 | 0.40 | 0.20 to 0.77 | 0.008 | 0.74 | 0.32 to 1.68 | 0.478 |
| P for trend | 0.018 | | | 0.024 | | | 0.011 | | | 0.358 | | |
| 5-Methyl-tetrahydrofolate (continuous), nmol/L | 0.54 | 0.34 to 0.85 | 0.008 | 0.54 | 0.34 to 0.87 | 0.011 | 0.48 | 0.29 to 0.78 | 0.003 | 0.69 | 0.37 to 1.26 | 0.228 |
| 5-Methyl-tetrahydrofolate (categorical), nmol/L |  |  |  |  |  |  |  |  |  |  |  |  |
| Q1 | Ref |  |  | Ref |  |  | Ref |  |  | Ref |  |  |
| Q2 | 0.92 | 0.52 to 1.62 | 0.775 | 0.91 | 0.51 to 1.62 | 0.752 | 0.90 | 0.50 to 1.61 | 0.728 | 0.93 | 0.45 to 1.88 | 0.831 |
| Q3 | 0.92 | 0.52 to 1.62 | 0.775 | 0.95 | 0.54 to 1.70 | 0.874 | 0.83 | 0.46 to 1.50 | 0.548 | 0.82 | 0.40 to 1.67 | 0.576 |
| Q4 | 0.42 | 0.21 to 0.79 | 0.009 | 0.40 | 0.20 to 0.77 | 0.008 | 0.37 | 0.18 to 0.73 | 0.005 | 0.69 | 0.29 to 1.58 | 0.381 |
| P for trend | 0.018 | | | 0.020 | | | 0.010 | | | 0.352 | | |
| Folic acid (continuous), nmol/L | 0.97 | 0.66 to 1.36 | 0.856 | 1.05 | 0.72 to 1.49 | 0.788 | 1.10 | 0.75 to 1.56 | 0.608 | 1.16 | 0.73 to 1.86 | 0.527 |
| Folic acid (categorical), nmol/L |  |  |  |  |  |  |  |  |  |  |  |  |
| Q1 | Ref |  |  | Ref |  |  | Ref |  |  | Ref |  |  |
| Q2 | 0.96 | 0.54 to 1.71 | 0.883 | 0.99 | 0.55 to 1.80 | 0.986 | 1.02 | 0.56 to 1.86 | 0.948 | 0.98 | 0.46 to 2.06 | 0.954 |
| Q3 | 0.88 | 0.49 to 1.57 | 0.655 | 1.00 | 0.55 to 1.83 | 0.998 | 1.03 | 0.56 to 1.90 | 0.916 | 0.94 | 0.45 to 1.98 | 0.879 |
| Q4 | 0.80 | 0.44 to 1.45 | 0.469 | 0.91 | 0.49 to 1.68 | 0.764 | 0.99 | 0.53 to 1.84 | 0.972 | 1.32 | 0.62 to 2.82 | 0.476 |
| P for trend | 0.337 | | | 0.661 | | | 0.791 | | | 0.543 | | |

Abbreviations: BMI, body mass index; CI, confidence interval; NAFLD, non-alcoholic fatty liver disease; OR, Odds Ratio.

^a^ Adjusted for gender, age, race/ethnicity, income-poverty ratio.

^b^ Adjusted for gender, age, race/ethnicity, income-poverty ratio, total energy, total cholesterol, smoking status.

^c^ Adjusted for gender, age, race/ethnicity, income-poverty ratio, total energy, total cholesterol, smoking status and BMI.

**eTable 2**. Associations of different folate forms with significant fibrosis(>8 kPa) among teenagers aged 12-19 years(n=549).

|  |  | | | **Adjusted model** | | | | | | | | |
| --- | --- | --- | --- | --- | --- | --- | --- | --- | --- | --- | --- | --- |
| **Folate** | **Unadjusted model** | | | **Model 1 ^a^** | | | **Model 2 ^b^** | | | **Model 3 ^c^** | | |
|  | **OR** | **CI (95%)** | ***P*** | **OR** | **CI (95%)** | ***P*** | **OR** | **CI (95%)** | ***P*** | **OR** | **CI (95%)** | ***P*** |
| Red blood cell folate (continuous), nmol/L | 0.19 | 0.06 to 0.68 | 0.009 | 0.23 | 0.06 to 0.93 | 0.035 | 0.27 | 0.07 to 1.12 | 0.065 | 0.26 | 0.06 to 1.15 | 0.069 |
| Red blood cell folate (categorical), nmol/L |  |  |  |  |  |  |  |  |  |  |  |  |
| Q1 | Ref |  |  | Ref |  |  | Ref |  |  | Ref |  |  |
| Q2 | 0.86 | 0.27 to 2.65 | 0.787 | 0.90 | 0.28 to 2.88 | 0.864 | 0.89 | 0.26 to 2.94 | 0.849 | 0.83 | 0.24 to 2.80 | 0.764 |
| Q3 | 0.86 | 0.27 to 2.65 | 0.787 | 1.09 | 0.32 to 3.56 | 0.890 | 1.26 | 0.37 to 4.24 | 0.708 | 1.25 | 0.37 to 4.26 | 0.714 |
| Q4 | 0.28 | 0.04 to 1.17 | 0.114 | 0.37 | 0.05 to 1.62 | 0.226 | 0.43 | 0.06 to 1.97 | 0.317 | 0.45 | 0.06 to 2.08 | 0.343 |
| P for trend | 0.144 | | | 0.345 | | | 0.517 | | | 0.560 | | |
| Serum total folate (continuous), nmol/L | 0.21 | 0.08 to 0.54 | 0.001 | 0.25 | 0.09 to 0.67 | 0.006 | 0.31 | 0.11 to 0.85 | 0.022 | 0.34 | 0.11 to 0.98 | 0.045 |
| Serum total folate (categorical), nmol/L |  |  |  |  |  |  |  |  |  |  |  |  |
| Q1 | Ref |  |  | Ref |  |  | Ref |  |  | Ref |  |  |
| Q2 | 0.53 | 0.18 to 1.43 | 0.223 | 0.48 | 0.15 to 1.34 | 0.171 | 0.50 | 0.16 to 1.44 | 0.210 | 0.48 | 0.15 to 1.41 | 0.192 |
| Q3 | 0.17 | 0.03 to 0.65 | 0.023 | 0.20 | 0.03 to 0.78 | 0.040 | 0.23 | 0.03 to 0.96 | 0.072 | 0.24 | 0.04 to 1.01 | 0.082 |
| Q4 | 0.17 | 0.03 to 0.65 | 0.023 | 0.21 | 0.03 to 0.87 | 0.054 | 0.24 | 0.03 to 1.01 | 0.080 | 0.29 | 0.04 to 1.30 | 0.139 |
| P for trend | 0.004 | | | 0.012 | | | 0.026 | | | 0.043 | | |
| 5-Methyl-tetrahydrofolate (continuous), nmol/L | 0.22 | 0.09 to 0.54 | 0.001 | 0.26 | 0.10 to 0.67 | 0.006 | 0.32 | 0.12 to 0.85 | 0.022 | 0.35 | 0.12 to 0.99 | 0.047 |
| 5-Methyl-tetrahydrofolate (categorical), nmol/L |  |  |  |  |  |  |  |  |  |  |  |  |
| Q1 | Ref |  |  | Ref |  |  | Ref |  |  | Ref |  |  |
| Q2 | 0.53 | 0.18 to 1.43 | 0.223 | 0.47 | 0.15 to 1.32 | 0.162 | 0.48 | 0.15 to 1.39 | 0.187 | 0.45 | 0.14 to 1.32 | 0.156 |
| Q3 | 0.17 | 0.03 to 0.65 | 0.023 | 0.20 | 0.03 to 0.80 | 0.044 | 0.24 | 0.04 to 1.00 | 0.078 | 0.25 | 0.04 to 1.05 | 0.090 |
| Q4 | 0.17 | 0.03 to 0.65 | 0.023 | 0.21 | 0.03 to 0.87 | 0.054 | 0.24 | 0.03 to 1.00 | 0.078 | 0.28 | 0.04 to 1.27 | 0.132 |
| P for trend | 0.004 | | | 0.013 | | | 0.020 | | | 0.044 | | |
| Folic acid (continuous), nmol/L | 0.92 | 0.38 to 1.79 | 0.835 | 0.88 | 0.34 to 1.82 | 0.766 | 0.93 | 0.36 to 1.91 | 0.858 | 0.88 | 0.35 to 1.80 | 0.762 |
| Folic acid (categorical), nmol/L |  |  |  |  |  |  |  |  |  |  |  |  |
| Q1 | Ref |  |  | Ref |  |  | Ref |  |  | Ref |  |  |
| Q2 | 4.78 | 0.55 to 1.71 | 0.048 | 4.28 | 1.04 to 29.07 | 0.072 | 4.99 | 1.15 to 35.05 | 0.053 | 4.66 | 1.07 to 30.84 | 0.065 |
| Q3 | 3.11 | 0.47 to 1.51 | 0.169 | 2.87 | 0.62 to 20.31 | 0.212 | 3.19 | 0.66 to 23.32 | 0.180 | 2.97 | 0.61 to 21.73 | 0.209 |
| Q4 | 2.05 | 0.42 to 1.39 | 0.413 | 1.91 | 0.35 to 14.39 | 0.472 | 2.26 | 0.40 to 17.80 | 0.377 | 2.26 | 0.40 to 17.70 | 0.376 |
| P for trend | 0.757 | | | 0.816 | | | 0.704 | | | 0.669 | | |

Abbreviations: BMI, body mass index; CI, confidence interval; OR, Odds Ratio.

^a^ Adjusted for gender, age, race/ethnicity, income-poverty ratio.

^b^ Adjusted for gender, age, race/ethnicity, income-poverty ratio, total energy, total cholesterol, smoking status.

^c^ Adjusted for gender, age, race/ethnicity, income-poverty ratio, total energy, total cholesterol, smoking status and BMI.

**eTable 3**.Associations of different folate forms with CAP and liver stiffness among teenagers aged 12-19 years(n=549)(mutually adjustment for 5-mTHF and Folic acid).

|  |  | | | **Adjusted model** | | | | | | | | |
| --- | --- | --- | --- | --- | --- | --- | --- | --- | --- | --- | --- | --- |
| **Folate** | **Unadjusted model** | | | **Model 1 ^a^** | | | **Model 2 ^b^** | | | **Model 3 ^c^** | | |
|  | **β** | **CI (95%)** | ***P*** | **β** | **CI (95%)** | ***P*** | **β** | **CI (95%)** | ***P*** | **β** | **CI (95%)** | ***P*** |
| **Associations of different folate forms with CAP** |  |  |  |  |  |  |  |  |  |  |  |  |
| 5-Methyl-tetrahydrofolate (continuous), nmol/L | -18.56 | -28.80 to -8.32 | <.001 | -13.69 | -27.26 to -6.34 | 0.002 | -14.13 | -28.98 to -7.86 | 0.001 | -19.74 | -30.60 to -8.87 | <.001 |
| 5-Methyl-tetrahydrofolate (categorical), nmol/L |  |  |  |  |  |  |  |  |  |  |  |  |
| Q1 | Ref |  |  | Ref |  |  | Ref |  |  | Ref |  |  |
| Q2 | -1.71 | -14.91 to 11.48 | 0.799 | -1.69 | -14.63 to 11.26 | 0.798 | -2.07 | -15.03 to 10.90 | 0.755 | -2.47 | -15.67 to 10.72 | 0.713 |
| Q3 | -2.43 | -15.62 to 10.76 | 0.718 | -1.29 | -14.34 to 11.75 | 0.846 | -3.49 | -16.64 to 9.66 | 0.603 | -3.99 | -17.48 to 9.50 | 0.562 |
| Q4 | -20.28 | -33.47 to -7.08 | 0.003 | -19.34 | -32.70 to -5.99 | 0.005 | -20.19 | -33.57 to -6.80 | 0.003 | -20.78 | -34.62 to -6.93 | 0.003 |
| P for trend | 0.004 | | | 0.009 | | | 0.005 | | | 0.005 | | |
| Folic acid (continuous), nmol/L | -2.44 | -10.48 to 5.61 | 0.553 | 0.298 | -7.71 to 8.30 | 0.942 | 0.745 | -7.26 to 8.75 | 0.855 | 4.22 | -3.93 to 12.37 | 0.311 |
| Folic acid (categorical), nmol/L |  |  |  |  |  |  |  |  |  |  |  |  |
| Q1 | Ref |  |  | Ref |  |  | Ref |  |  | Ref |  |  |
| Q2 | -1.39 | -14.71 to 11.93 | 0.838 | -0.216 | -13.38 to 12.95 | 0.974 | 0.18 | -12.97 to 13.34 | 0.978 | 1.88 | -11.29 to 15.05 | 0.780 |
| Q3 | -5.36 | -18.68 to 7.96 | 0.430 | -1.82 | -15.08 to 11.43 | 0.788 | -1.11 | -14.36 to 12.14 | 0.870 | 2.35 | -11.18 to 15.88 | 0.733 |
| Q4 | -6.87 | -20.19 to 6.45 | 0.313 | -2.55 | -15.84 to 10.75 | 0.707 | -1.74 | -15.06 to 11.58 | 0.798 | 1.94 | -11.64 to 15.51 | 0.780 |
| P for trend | 0.253 | | | 0.668 | | | 0.762 | | | 0.711 | | |
| **Associations of different folate forms with liver stiffness** |  | | |  | | |  | | |  | | |
| 5-Methyl-tetrahydrofolate (continuous), nmol/L | -1.14 | -1.75 to -0.53 | <.001 | -1.07 | -1.70 to -0.44 | 0.001 | -0.73 | -1.35 to -0.11 | 0.021 | -0.76 | -1.39 to -0.12 | 0.020 |
| 5-Methyl-tetrahydrofolate (categorical), nmol/L |  |  |  |  |  |  |  |  |  |  |  |  |
| Q1 | Ref |  |  | Ref |  |  | Ref |  |  | Ref |  |  |
| Q2 | -0.47 | -1.26 to 0.32 | 0.248 | -0.50 | -1.29 to 0.29 | 0.216 | -0.33 | -1.09 to 0.43 | 0.394 | -0.40 | -1.17 to 0.38 | 0.316 |
| Q3 | -0.97 | -1.76 to -0.18 | 0.017 | -0.89 | -1.68 to -0.09 | 0.029 | -0.56 | -1.33 to 0.21 | 0.154 | -0.63 | -1.42 to 0.16 | 0.118 |
| Q4 | -1.02 | -1.81 to -0.23 | 0.012 | -0.91 | -1.72 to -0.09 | 0.029 | -0.67 | -1.45 to 0.12 | 0.097 | -0.74 | -1.55 to 0.07 | 0.074 |
| P for trend | 0.005 | | | 0.017 | | | 0.078 | | | 0.074 | | |
| Folic acid (continuous), nmol/L | -0.10 | -0.59 to 0.38 | 0.673 | -0.06 | -0.55 to 0.42 | 0.799 | -0.06 | -0.52 to 0.41 | 0.816 | 0.08 | -0.40 to 0.56 | 0.751 |
| Folic acid (categorical), nmol/L |  |  |  |  |  |  |  |  |  |  |  |  |
| Q1 | Ref |  |  | Ref |  |  | Ref |  |  | Ref |  |  |
| Q2 | 0.80 | 0.01 to 1.59 | 0.049 | 0.76 | -0.04 to 1.55 | 0.063 | 0.76 | -0.01 to 1.52 | 0.052 | 0.85 | 0.08 to 1.62 | 0.030 |
| Q3 | 0.16 | -0.63 to 0.95 | 0.686 | 0.18 | -0.62 to 0.98 | 0.654 | 0.20 | -0.56 to 0.98 | 0.596 | 0.39 | -0.40 to 1.18 | 0.337 |
| Q4 | 0.05 | -0.74 to 0.84 | 0.901 | 0.14 | -0.67 to 0.94 | 0.739 | 0.14 | -0.63 to 0.91 | 0.722 | 0.32 | -0.48 to 1.11 | 0.434 |
| P for trend | 0.710 | | | 0.915 | | | 0.927 | | | 0.739 | | |

Abbreviations: BMI, body mass index; CI, confidence interval; NAFLD, non-alcoholic fatty liver disease; OR, Odds Ratio.

^a^ Adjusted for gender, age, race/ethnicity, income-poverty ratio.

^b^ Adjusted for gender, age, race/ethnicity, income-poverty ratio, total energy, total cholesterol, smoking status.

^c^ Adjusted for variables in model 2, plus mutually adjustment for 5-mTHF and Folic acid.
